# Magnetoelectric Coupling in Room Temperature Multiferroic Ba_2_EuFeNb_4_O_15_/BaFe_12_O_19_ Epitaxial Heterostructures Grown by Laser Ablation

**DOI:** 10.3390/nano13040761

**Published:** 2023-02-17

**Authors:** Thameur Hajlaoui, Catalin Harnagea, Alain Pignolet

**Affiliations:** Centre Énergie, Matériaux et Télécommunications, Institut National de la Recherche Scientifique, 1650 Boulevard Lionel-Boulet, Varennes, QC J3X 1P7, Canada

**Keywords:** epitaxial thin films, room temperature multiferroic, nanocomposite, magnetoelectric coupling, multifunctional heterostructures

## Abstract

Multiferroic thin films are a promising class of multifunctional materials, since they allow the integration of multiple functionalities within a single device. In order to overcome the scarcity of single phase multiferroics, it is crucial to develop novel multiferroic heterostructures, combining good ferroelectric and ferromagnetic properties as well as a strong coupling between them. For this purpose, Ba_2_EuFeNb_4_O_15_/BaFe_12_O_19_ multiferroic magnetoelectric bilayers have been epitaxially grown on niobium doped SrTiO_3_ (100) single crystal substrates by pulsed laser deposition. The simultaneous presence of both ferroelectric and magnetic properties—due, respectively, to the Ba_2_EuFeNb_4_O_15_ and BaFe_12_O_19_ components—was demonstrated at room temperature, attesting the multiferroic nature of the heterostructure. More interestingly, a strong magnetoelectric coupling was demonstrated (i) by manipulating the ferroelectric properties via an external magnetic field, and conversely, (ii) by tuning the magnetic properties via an external electric field. This strong magnetoelectric coupling shows the high interdependence of both ferroic orders in the Ba_2_EuFeNb_4_O_15_/BaFe_12_O_19_ heterostructure, mediated by elastic (epitaxial) strain at the interfaces.

## 1. Introduction

Multiferroics (MF) are materials that exhibit at least two ferroic properties (ferroelectricity, ferromagnetism, ferroelasticity, etc.) simultaneously [1,2,3]. The presence of different ferroic orders within the same material is of great fundamental interest due to the rich physics involved that generally leads to couplings between them [1,4]. In particular, the magnetoelectric (ME) coupling appears to be the most interesting one for its potential use in a variety of applications [4,5], representing a challenging, yet very exciting research field. In short, the magnetoelectric effect is defined as a change in polarization upon the application of an external magnetic field (direct effect) or a change in magnetization upon the application of an external electric field (converse effect) [1,2].

Due to the scarcity of single phase multiferroic magnetoelectric (MF-ME) materials in nature [6] and the practical importance for the materials to possess these properties at room temperature (RT), many strategies have been adopted to develop novel materials exhibiting room temperature magnetoelectric properties. One of the most efficient approaches is to synthesize, instead of single phase multiferroics, composite materials with multiferroic properties and a strong magnetoelectric coupling [7,8]. In these material systems, the ferroelectric and the ferromagnetic phases are distinct, and are elastically coupled to each other at their interface [9,10].

In terms of applications in real devices, MF-ME composites have shown promises in various domains such as energy harvesting, current sensing, microwave and millimeter-wave devices as well as medical applications [9,10,11,12]. In addition, the combination of different phases can significantly improve the electronic properties of the resulting composite material [13,14]. In particular, bilayer MF-ME composite thin films have been synthesized and studied for electrically written and magnetically read magnetoelectric memory devices with a high density of information storage, high thermal stability and low energy consumption [9,10,15,16].

Since 2009, a number of studies have been dedicated to multiferroic composite ceramics based on the material with tetragonal tungsten bronze structure Ba_2_LnFeNb_4_O_15_ (TTB-Ln, with Ln = Eu^3+^, Nd^3+^ and Sm^3+^) [17,18,19,20]. While the ferroelectricity in these ceramics is due to the TTB-Ln phase, their magnetic properties were shown to originate from *spontaneous* formation during their synthesis of a magnetic barium hexaferrite BaFe_12_O_19_ (BaFO) secondary phase, revealing the composite nature of these multiferroics. The spontaneous formation of the magnetic phase within the ferroelectric matrix ensures the chemical compatibility of the different phases, an important and well-known issue in composites [21,22]. Another important aspect of these TTB-Ln/BaFO multiferroic composites is their high ferroelectric and magnetic Curie temperatures, estimated, respectively, at ≈440 K [17] and ≈800 K [23,24]. In addition, the ˋhardˊ magnetic BaFO phase is very well known and studied as it is used in several applications, including magnetic recording and microwave applications [25,26,27]. It is worth mentioning that Al-doped BaFO is demonstrated to be multiferroic and magnetoelectric but in the form of a ceramic [28,29], even though the coercive field of the resulting ceramic is considerably reduced due to the doping.

Motivated by the works on TTB-Ln ceramics, we succeeded in synthesizing the RT multiferroic nanocomposite with a tetragonal tungsten bronze structure Ba_2_EuFeNb_4_O_15_/BaFe_12_O_19_ (TTB-Eu) in a thin films form using pulsed laser deposition (PLD) [30]. Various techniques have been used to synthesize high quality films of functional materials [31,32,33,34]. Among them, PLD was recognized for its simple setup and its versatility, in addition to its deposition energetics and kinetics, allowing for high quality films of complex stoichiometry and crystal structure such as, for instance, multicomponent complex oxides [34,35,36]. Similarly to ceramics, the multiferroic nature of these films was found to be due to the presence of BaFO nanoregions embedded in the ferroelectric TTB-Eu matrix. Since the films obtained by sputtering [31], using the same ceramic targets containing embedded BaFO nanoregions, have not shown multiferroic properties at room temperature, the presence of the BaFO phase in our PLD-grown films is thought to be mainly explained by the stoichiometric transfer provided by the PLD. By optimizing the deposition conditions, we have achieved high-quality epitaxial multiferroic thin films with ferroelectric properties which are considerably enhanced compared to ceramics [37,38]. Nevertheless, their magnetic properties were weak even after varying the nature of Ln ions (chemical composition) [39,40], making the study of the magnetoelectric coupling in these films very challenging. 

As a next step to enhance the magnetism in order to be able to investigate the magnetoelectric coupling, we synthesized epitaxial Ba_2_EuFeNb_4_O_15_/BaFe_12_O_19_ (TTB-Eu/BaFO) bilayers. In this report, we discuss the multiferroic properties of these heterostructures and we show that they exhibit a strong magnetoelectric coupling at room temperature. This coupling is evidenced first by studying the effect of an applied magnetic field on the ferroelectric properties, and second by demonstrating the effect of an electric field on the magnetic properties of the synthesized heterostructures. 

## 2. Materials and Methods

Although BaFe_12_O_19_ thin films have been synthesized using a variety of methods [41,42,43], Ba_2_EuFeNb_4_O_15_ films have only been deposited by sputtering [31] and by pulsed laser deposition because of their complex structure [37,38,39,40,44]. Therefore, in order to prepare the Ba_2_EuFeNb_4_O_15_/BaFe_12_O_19_ heterostructured nanocomposite, BaFe_12_O_19_ (BaFO) and Ba_2_EuFeNb_4_O_15_ (TTB-Eu) thin films have been sequentially grown on single crystalline Nb-doped SrTiO_3_ (100) (NSTO) substrates using pulsed laser deposition (PLD). NSTO was used due to its electric conductivity, allowing its use as the bottom electrode for electromechanical characterization, its stability at high temperature (≥750 °C), as well as its crystal structure enabling the epitaxial growth of both BaFO and TTB-Eu films. Prior to each deposition, the NSTO substrates have been chemically treated using acetone and isopropyl alcohol before being heated in an oxygen atmosphere at 1000 °C for 1 h to ensure high-quality surfaces. In the first stage, BaFO films were deposited using a stoichiometric ceramic target of barium hexaferrite BaFe_12_O_19,_ synthesized through a solid-state reaction route using BaCO_3_ and Fe_2_O_3_ precursors. The density of the target synthesized at optimized conditions was higher than 90% of the theoretical density, suitable for laser ablation. This target was ablated using an energetic pulsed KrF excimer laser beam with wavelength *λ* = 248 nm and pulse duration of 25 ns. The area of the focused laser spot ablating the rotating target was 2 mm^2^ resulting in a laser fluency on the target of 2.8 J/cm^2^. In order to ablate the target more uniformly, the laser beam was scanned across the rotating target. The repetition rate of the laser pulses during deposition was set at 20 pulses/second, and the substrate temperature was set at 750 °C. The obtained amorphous films were annealed, ex situ, at the optimized temperature of 850 °C to achieve the crystallization of the BaFO phase and obtain the desired crystalline epitaxial BaFO films. The thickness of the epitaxial BaFO films was estimated at around 20 nm. In the second stage, TTB-Eu films were deposited using PLD on the epitaxial BaFO films with the same deposition conditions as for the BaFO films, using a TTB-Eu ceramic target with a density higher than 96% of the theoretical density [17]. Although they had been deposited at a substrate temperature of 750 °C, the PLD-grown TTB–Eu films obtained were amorphous. The [amorphous TTB-Eu films /epitaxial BaFO films] heterostructures were then annealed at the optimized temperature of 800 °C to epitaxially crystalize the TTB-Eu films, without affecting (in terms of microstructure and crystalline quality) the layers of BaFO beneath. The thickness of TTB-Eu crystalline films was determined to be around 180 nm, for a total thickness of the heterostructures of approximately 200 nm. The reduced thickness of BaFO (20 nm) was chosen due to two main reasons: (i) A thickness of 20 nm of BaFO is enough to ensure significant magnetic properties in the studied bilayers and (ii) to ensure an epitaxial growth of TTB-Eu layer governed by the underlying substrate crystal structure through the magnetic phase. 

The presence and the quality of the crystalline phases were investigated using X-ray diffraction, performed using a 4-circle X-ray diffractometer especially designed to study epitaxial thin films (PANalytical X-Pert PRO MRD). The microstructure, the local electromechanical properties, the surface potential (SP) and the microscopic magnetic response of the TTB-Eu/BaFO bilayer thin films were characterized using a modified atomic force microscope (AFM). The investigations were performed using a DI-enviroscope AFM (Bruker, Santa Barbara, CA, USA) modified to be able to perform piezoresponse measurements as well as simultaneous Kelvin Probe Force Microscopy (KPFM) and Magnetic Force Microscopy (MFM). Piezoresponse force microscopy (PFM) was used to image and manipulate the films ferroelectric polarization at the nanoscale. For PFM, an alternating voltage of 1 V at 20 kHz was applied between the conductive tip and the NSTO substrate, and the surface-induced piezoelectric vibrations were detected using a lock-in amplifier from Signal Recovery (model 7265, Wokingham, UK). MFM was performed to investigate the magnetic response of the TTB-Eu/BaFO bilayered system. In order to eliminate the electrostatic effects on the magnetic signal, the MFM measurements were performed with SP compensation. To achieve this, MFM and KPFM were performed simultaneously. KPFM was implemented using a PLLproII controller from RHK Technology. A double modulation scheme was used, mechanically oscillating the cantilever at its first (fundamental) resonance (around 55 kHz, tip-dependent) for topography imaging, while the second resonance (≈310 kHz) was used for electrostatic modulation [45] and compensation. Both topography and SP were measured simultaneously, in a single pass. For KPFM-compensated MFM, the usual double-pass technique was used (‘lift mode’), with the KPFM feedback being activated continuously both in the first pass (topography and SP detection) and the second pass (detection of the magnetic interaction via the resonance frequency shift with the AFM tip raised 40 nm above the surface, detected during the first pass and with the electrostatic interaction being nullified by the KPFM feedback loop). The lateral resolution of the KPFM images when using lift mode was inadequate for KPFM imaging and KPFM is only used for electrostatic compensation during MFM. The amplitude of the AC excitation applied for KPFM was only 0.5 V, due to the use of the second resonance [46,47]. All measurements, PFM, KPFM and MFM were performed with magnetic tips attached to medium-stiff cantilevers, having a Co/Cr coating (MESP from Bruker, Santa Barbara, CA, USA), suitable for both electric and magnetic measurements. All XRD, AFM, PFM, KPFM and MFM measurements were performed at room temperature.

Finally, the heterostructures magnetic properties were assessed via magnetic hysteresis loop measurements, describing the variation in the macroscopic magnetization as a function of an applied magnetic field. The measurements were performed at room temperature using an EV9 vibrating sample magnetometer (VSM, -Lowell, MA, USA), with the magnetic field being applied in the plane of the measured samples. An average of 50 measurements per magnetic field value provided an absolute sensitivity of about 10^−6^ emu. The magnetic response of the sample holding rods and bare substrates were also measured and subtracted in order to extract the magnetic signal originating solely from the studied heterostructure.

## 3. Results

### 3.1. Topographic and Structural Characterization

**Figure 1:**
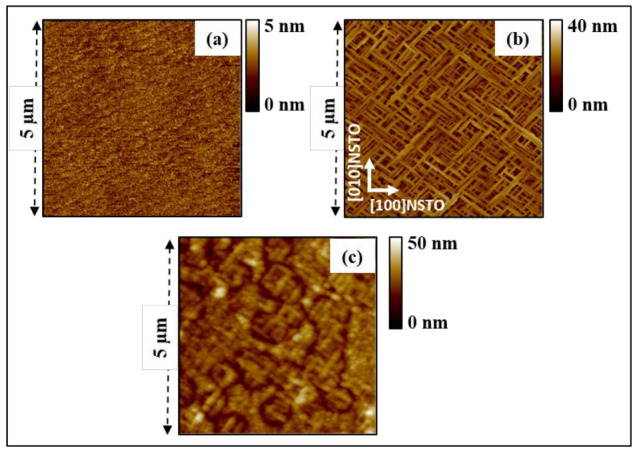
AFM images representing the topography of the surface of (**a**) NSTO (001) substrate; (**b**) of BaFO thin film deposited on top of a NSTO substrate and annealed at 850 °C while (**c**) shows the surface topography of the TTB-Eu/BaFO/NSTO heterostructure after annealing at 800 °C.

**Figure 2:**
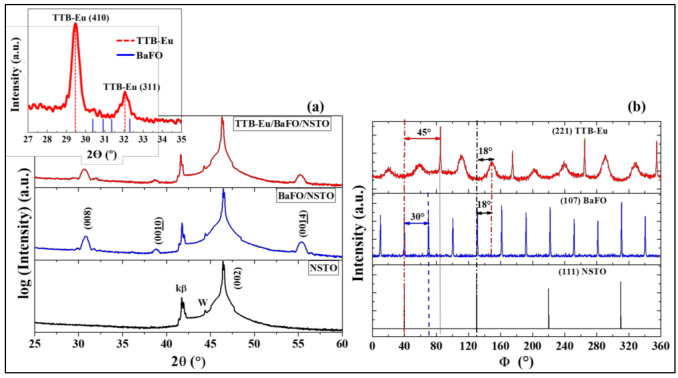
Structural characterization of TTB-Eu/BaFO/NSTO (001) heterostructured films: (**a**) X-ray diffractograms of NSTO substrate, BaFO films and TTB-Eu/BaFO heterostructure; (**b**) Φ-scan measurements of the {221} planes of TTB-Eu films, as well as of the {107} planes of the BaFO intermediate magnetic layer and the {111} planes of the single crystalline NSTO (001) substrate. The inset of figure (**a**) shows the in-plane X-ray diffractogram of the heterostructure where the red lines indicate the positions of the TTB-Eu peaks and the blue lines denote the peaks positions of the BaFO phase, allowing the characterization of the crystallization and the orientation of the TTB-Eu films.

The topography and the microstructure of the substrates and films surfaces were studied using AFM measurement and are shown in Figure 1. The topographic image shown in Figure 1a reveals a very smooth surface with a measured RMS roughness ≈0.6 nm, indicating an excellent surface quality of the substrate. A structure of terraces is observed with an average terrace width around 215 nm. The topography image in Figure 1b shows the surface of the BaFO films annealed at 850 °C. The microstructure of these films shows a pattern of highly anisotropic grains, forming a network of nanorods lying in the plane of the film, with lengths varying between 0.5 µm and 2 µm, and width of around 100 nm. The nanorod axes (i.e., the fast growth directions of the grains) are parallel to the [110] or [1–10] of the NSTO substrate (Figure 1b). The BaFO layer is characterized by an RMS roughness of ≈8.3 nm. Similar grain microstructure has been observed for barium and strontium hexaferrite thin films deposited on SrTiO_3_ (100) and a-Al_2_O_3_ [48,49]. Figure 1c depicts the surface topography of the TTB-Eu/BaFO/NSTO heterostructure annealed at 800 °C in order to crystallize the amorphous as-deposited TTB-Eu layer, which is characterized by an RMS roughness of ≈3.15 nm. Further analysis (Figure S1 in Supplementary Materials) demonstrates that the TTB-Eu crystallization annealing at 800 °C does not affect the microstructure and the phase purity of the underlying BaFO layer. Thereby, the observed variation of the microstructure at the surface of the bilayer upon annealing is related only to the crystallization of the TTB-Eu phase into an epitaxial film, as shown by a detailed X-ray diffraction analysis (Figure 2). In addition, the ferroelectric TTB-Eu layer filling the space between the magnetic BaFO nanorods increases the area of the interface between the two layers. This does enhance the interfacial interactions between the two components of this multiferroic composite system, enhancing the magnetoelectric coupling [50,51,52] between the ferroelectric (TTB-Eu) and the magnetic (BaFO) components.

In order to characterize the crystalline structure of the synthesized multiferroic system, conventional θ/2θ diffraction, in-plane diffraction and Φ-scan measurements were used and are presented in Figure 2. For an accurate identification of the film diffraction peaks, θ/2θ measurement was also performed for an NSTO (001) bare substrate (black curve in Figure 2a. In the NSTO (001) substrate diffractogram, only the (200) diffraction peak of the single crystalline substrate can be identified at 2θ ≈ 46.43°, which is accompanied by its k_β_ reflection at 2θ ≈ 41.73° and a small peak attributed to the X-ray tungsten tube contamination at 2θ ≈ 44.39°. In the same figure, the blue diffractogram of the BaFO layer on NSTO (001) annealed at 850 °C shows that for the BaFO films only the (00l) peaks of the BaFO hexagonal structure are observed, thus attesting an oriented growth of the BaFO film with their c-axis perpendicular to the substrate surface. No additional peaks are observed in the θ/2θ diffractogram of the annealed TTB-Eu/BaFO/NSTO (001) heterostructure (red curve), suggesting that either the films TTB-Eu are amorphous; or the (00l) peaks of the crystalline TTB-Eu ferroelectric phase overlap with the very high intensity (h00) single crystal substrate peaks, or with peaks of the underlying BaFO film, making this measurement configuration inadequate to study, or even evidence the TTB-Eu crystallographic structure and orientation. Therefore, we adopted the in-plane X-ray diffraction geometry [53] with a grazing incidence angle alpha of 0.4°, which indeed demonstrated that the TTB-Eu films was well crystallized and allowed a detailed investigation of the crystal structure and orientation of the ferroelectric phase (see the inset of Figure 2a): The peaks observed clearly corresponded to the TTB-Eu crystal structure, as shown by the red lines (JCPDS reference file #00-058-0648), while the peaks corresponding to the underlying BaFO phase given by the blue lines (JCPDS reference file # 00-007-0276) are present but barely visible. This measurement demonstrates that the peaks obtained through the in-plane diffraction perfectly correspond to the TTB-Eu phase, evidencing the crystallization of this phase after the annealing at 800 °C. In addition, the intensity of the (410) peak (representing the family of planes perpendicular to the films surface) is much higher than that of the (311) peak (representing the family of planes with the highest intensity of TTB-Eu structure in its polycrystalline form according to the JCPDS # 00-058-0648). Thus, it can be inferred that the TTB-Eu film is mainly oriented with the c-axis perpendicular to the substrate surface (i.e., c-oriented), suggesting that a large fraction of the film is epitaxially grown on the underlying BaFO film, itself epitaxially grown onto the NSTO single crystalline substrate. 

In order to elucidate the details of the epitaxial growth between the substrate, BaFO and TTB-Eu structures, Φ-scans were performed as depicted in Figure 2b.

#### 3.1.1. Epitaxial Growth of BaFO and TTB-Eu on NSTO (001) Substrate

The in-plane orientation of the epitaxial BaFO films with respect to the NSTO crystal structure was studied by performing Φ-scan measurements of the {107} planes of the BaFO lattice (2θ = 32.29° and Ψ = 33.38°) and of the {111} planes of the NSTO substrate (2θ = 39.98° and Ψ = 54.74°) as shown in Figure 2b. While NSTO{111} is represented by four peaks, 90ᵒ apart, due to its cubic structure, the {107} BaFO is represented by 12 peaks every 30° over the full 360ᵒ range of the angle Φ, which can be divided in two groups. The first group is formed by four peaks which are situated at the same Φ angles as the underlying NSTO peaks, indicating that the intersections of respectively the {111} NSTO planes and the {107} BaFO planes with the {001} NSTO planes (which are parallel to {001} BaFO planes) are parallel (Figure S2 in Supplementary Materials). In this configuration, the epitaxial relationship is given by:

BaFO (001)||NSTO (001); [100] BaFO||[410] NSTO and [010] BaFO||[−110] NSTO.

**Figure 3:**
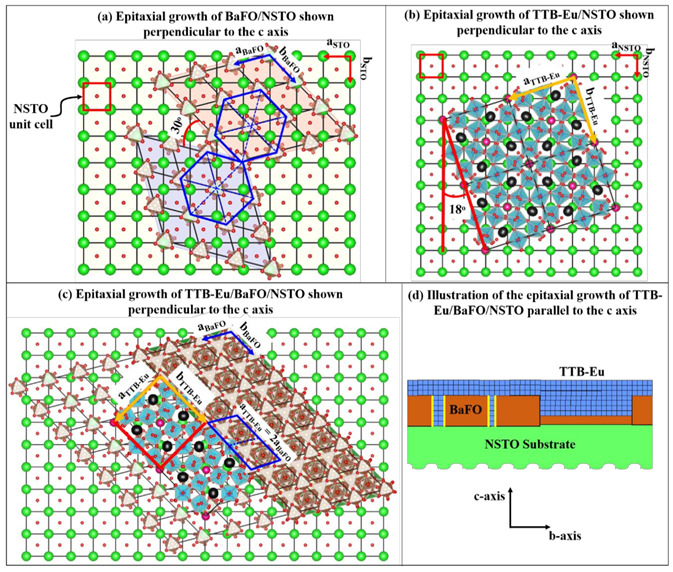
Crystallographic model illustrating the epitaxial relationships: (**a**) illustration of the epitaxial growth of the BaFO on the NSTO structure, where the two different in-plane orientations of the BaFO unit cells with respect to the NSTO crystal structure are shown, azimuthally separated by 30°. (**b**) Illustration of the epitaxial growth of the TTB-Eu directly on NSTO substrate, showing an in-plane rotation of 18° of the TTB-Eu unit cell with respect to the substrate lattice. This in-plane orientation corresponds to the eight peaks with low intensity observed in the red diffractogram in the Figure 2b. (**c**) Illustration of the epitaxial growth of TTB-Eu on top of an epitaxial BaFO/NSTO heterostructure. (**d**) Cross-sectional sketch shown along the [100] direction of NSTO illustrating the different variants of epitaxial growth of the TTB-Eu/BaFO/NSTO, with TTB-Eu growing both on top of BaFO or directly on top of NSTO, or possibly also on top a very thin BaFO layer, thin enough for the epitaxy to be determined using the underlying substrate crystal structure. The yellow lines illustrate the presence of a lateral epitaxial matching along the c-axis between the c_BaFO_ and stack of 6 × c_TTB-Eu_, implying an additional coupling between the TTB-Eu and BaFO which is benefic for the magnetoelectric coupling.

This epitaxial growth mode of BaFO is illustrated in Figure 3a where the BaFO unit cells are drawn in orange color. The BaFO unit cell is thus under an anisotropic compressive strain due to a lattice mismatch of ≈ +5% calculated as the relative difference between a_BaFO_ (or b_BaFO_) and a_NSTO_ $\times \sqrt{2}$ (diagonal of a face of the NSTO unit cell) along one crystallographic axis and a lattice mismatch of ≈ +7% (calculated as the relative difference between a_BaFO_ and a_NSTO_ $\times \sqrt{17}$ (diagonal of a block of 4 × NSTO unit cells placed in-line)) along the second crystallographic axis. Since there are four such possible equivalent configurations, the number of peaks in this group is four. The second group of peaks are located at an angle of ± 30° with respect to the NSTO peaks, indicating a 30° in-plane rotation of the BaFO unit cell compared to the NSTO lattice. This epitaxial growth mode of BaFO is illustrated by the violet-colored unit cells in Figure 3a. A compressive strain related to a lattice mismatch of ≈ +7% (between both a_BaFO_, b_BaFO_ and a_NSTO_ $\times \sqrt{17}$ (diagonal of 4 × NSTO unit cells placed in-line)) is obtained in this orientation with an epitaxial relationship:

BaFO (001)||NSTO (001); [100] BaFO||[410] NSTO and [010] BaFO||[−140] NSTO.

The number of peaks in this group is eight, since there are eight possible equivalent relative orientations of BaFO with respect to NSTO lattice. These two epitaxial growth mode of BaFO are present at the interfaces between the BaFO and the substrate as exhibited in the cross-section cartoon of the Figure 3d that depicts the different interfaces present between the substrate and the different components of the studied heterostructure.

To determine the in-plane orientation of the TTB-Eu lattice and unit cells, Φ-scan measurement was performed after positioning the goniometer at 2θ = 30.27° and Ψ = 41.7°, corresponding to the {221} planes of the TTB-Eu films. This Φ-scan is shown in Figure 2b, together with those of the {107} BaFO planes and {111} NSTO planes. Twelve peaks were obtained for the TTB-Eu film over the full 360° range, which can again be explained by considering two groups of peaks: The first group was formed by the eight relatively broad peaks that were shifted by an angle of 18° with respect to the (111) NSTO planes. In this configuration the TTB-Eu unit cells were rotated in-plane by an angle of 18° with respect to the NSTO lattice, corresponding to a lattice mismatch of ≈+0.8% and a slight compressive strain.

The epitaxial relationship with the substrate is described by:

TTB-Eu (001) || NSTO (001); [100] TTB-Eu || [3 -1 0] NSTO and

[010] TTB-Eu || [130] NSTO.

Since there are eight equivalent orientations respecting these epitaxial relationships, the number of peaks in this group is eight, as was explained in more detail in our previous work [30]. Because of the microstructure of the BaFO layer (see above), some amount of TTB-Eu comes in contact with the substrate and grows epitaxially on it, as illustrated in the cross-sectional sketch of Figure 3d. Usually, the Φ-scan measurements are used to determine the in-plane orientation of epitaxial films, whereby the peak broadening is indicative of the in-plane mosaicity (i.e., the angular range that the in-plane orientation can assume around the ideal epitaxial orientation). Thus, the peak broadening can be attributed to the presence of an in-plane mosaicity in the epitaxial growth TTB-Eu on NSTO, that is mainly caused by the presence of the BaFO structure as well as the annealing during the heterostructure synthesis. 

#### 3.1.2. Epitaxial Growth of TTB-Eu on BaFO Layer

The second group of (221) TTB-Eu peaks was composed of four sharp peaks situated at 45ᵒ from the subsets of (107) BaFO peaks that coincide with the (111) NSTO peaks, as shown in Figure 2b. Consequently, the TTB-Eu unit cell was rotated in-plane by 45° with respect to the NSTO crystal structure, as depicted in Figure 3b and Figure S2 in Supplementary Materials. Thus, this component of the TTB-Eu film was induced by the presence of the BaFO film and was under a compressive strain due to a lattice mismatch of ≈+6%, as calculated from the a_TTB-Eu_ and 2 × a_BaFO_ values. Therefore, the number of peaks in this group is the same as that of the first group of BaFO peaks mentioned above, i.e., four. In addition, the particular BaFO microstructure (that was claimed above to be beneficial for magnetoelectric coupling) implies the presence of a lateral epitaxial matching along the c-axis between the c_BaFO_ and stack of 6 × c_TTB-Eu_ (illustrated by the yellow lines in Figure 3d at the lateral interface between the TTB-Eu film and the BaFO film). In this case, low positive lattice mismatch of ≈+2% was calculated, evidencing an additional compressive strain applied by the BaFO structure on the TTB-Eu structure along the c-axis. Thus, an additional elastic coupling should be present between the ferroelectric and the magnetic components in the studied composite multiferroic system. The epitaxial relationship between the TTB-Eu and BaFO layers is summarized as:

TTB-Eu (001)||BaFO (001); [100] TTB-Eu||[210] BaFO and [010] TTB-Eu||[010] BaFO

### 3.2. Multiferroic Properties

**Figure 4:**
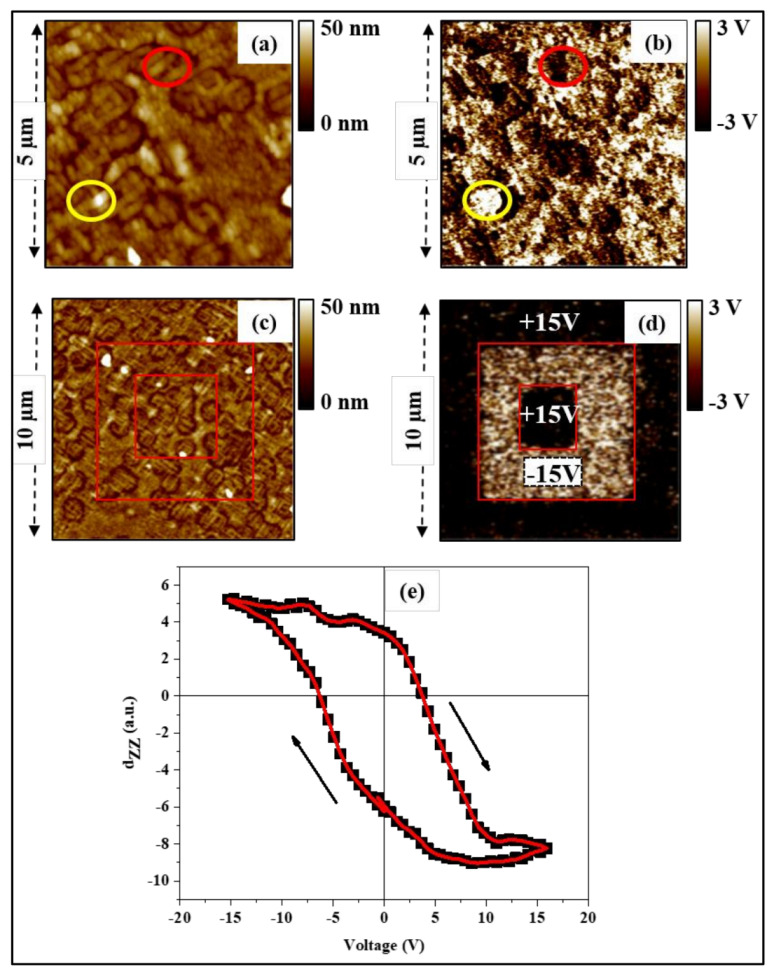
Microelectromechanical characterization of the TTB-Eu/BaFO heterostructure: (**a**) and (**c**) represent the topography of the surface; (**b**) and (**d**) show respectively the grown out-of-plane piezoelectric response and the switching behavior of the local polarization obtained by applying a DC voltage of ±15V. The red and yellow circles indicate two different regions with the out-of-plane polarization being oriented in opposite directions. The region circled in yellow is attributed to regions where the polarization is upward oriented while the region circled in red is associated with downward oriented polarization. (**e**) Hysteresis loop describing the variation of the longitudinal piezoelectric coefficient (d_ZZ_) as a function of an applied voltage bias, confirming the ferroelectric behavior of the synthesized heterostructure.

To investigate the local electromechanical properties of the synthesized heterostructure and get space-resolved information about their ferroelectric properties at room temperature, we performed PFM measurements. While Figure 4a,c displays the topography of the surface which is consistent with the previous discussion, Figure 4b,d shows the out-of-plane piezoelectric response (mixed signal, i.e., amplitude × cos(phase)) detected at the films surface. In the as-grown domain image (Figure 4b), two principal contrast levels are observed; while the bright contrast is attributed to polar regions where the out-of-plane component of the polarization is upward oriented (bottom-to-top or substrate to free surface, e.g., see the region circled in yellow), the dark contrast is associated with polar regions where the out-of-plane component of polarization is downward oriented (e.g., see the region circled in red). Careful comparison of the images in Figure 4a,b shows that the piezoelectric contrast has no correlation with the topography, excluding any possible topographic cross-talk in the PFM images. This comparison confirms that the contrast observed in Figure 4b is due to the presence of local polarization at the surface of the films.

The poling and switching of local polarization are evidenced by applying an external DC voltage via the PFM conductive tip while scanning the surface, as shown in Figure 4d. In this experiment, different domains were created by successively writing three concentric square patterns of 10 × 10 μm^2^, 6 × 6 μm^2^ and 3 × 3 μm^2^ areas on the film surface with +15 V, −15 V and +15 V DC voltages, respectively. The ferroelectric domains were then imaged with out-of-plane PFM. The comparison of Figure 4c,d demonstrates that the applied voltage does not affect the topography, attesting the high chemical and mechanical stability of the surface of the film. More importantly, it can be seen from the Figure 4d that two contrast levels can be created on the film’s surface, which demonstrates the writing of domains with polarization oriented up and/or down perpendicular to the surface plane. The good uniformity of the bright and dark domains demonstrates the 180° switching of the polarization, back and forth, at the film surface with the applied voltage [54]. This result demonstrates the good switching behavior of the ferroelectric nanocomposite and suggests that this heterostructure could be promising for memory devices that require the polarization to switch between two opposite states (180° switching) [9].

To further illustrate the ferroelectric nature of the studied system, we evaluated the variation of the longitudinal piezoelectric coefficient (d_ZZ_) [55,56] while cycling the external voltage applied to the PFM tip fixed above selected locations on the sample surface. The dependence of d_ZZ_ on the applied bias is described using a hysteresis loop as shown in Figure 4e. The well-saturated character of the hysteresis loop proves that the polarization can be reversibly switched between two opposite stable polarization states, and thus clearly confirms the ferroelectric nature of the TTB-Eu/BaFO system investigated.

**Figure 5:**
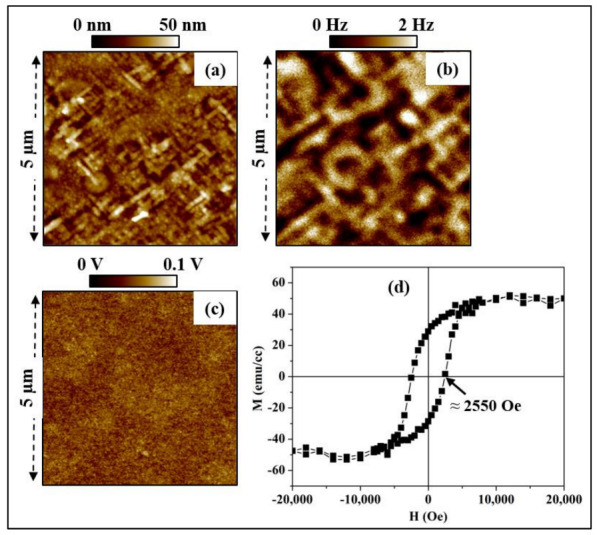
Microscopic magnetic properties of TTB-Eu/BaFO system: (**a**) topography of the surface, (**b**) magnetic response and (**c**) surface potential obtained using KPFM measurements. (**d**) Magnetic hysteresis loop M-H representing the variation of the macroscopic magnetization (M) as a function of magnetic field (H) applied in the plane of the samples.

The magnetic properties of the synthesized heterostructure TTB-Eu/BaFO were first studied microscopically using MFM [56]. Figure 5a–c shows, respectively, the topography, the magnetic response and the surface potential (all three acquired simultaneously) of the studied system. The different contrast levels observed in Figure 5b are attributed to magnetic regions with the magnetization oriented in different directions [57]. Note that the absence of any correlation between the topography or the surface potential and the MFM image is a good indication of the absence of artifacts, confirming that the contrast observed in Figure 5b is solely due to magnetic domains detected at the sample surface. The average magnetic domain length is estimated to be ~1 µm, consistent with the large magnetic coercive field measured macroscopically (Figure 5d) [57,58]. The very weak contrast observed in the surface potential image shown in Figure 5c (lower than 0.1 V) evidences the absence of any other non-magnetic force (e.g., electrostatic force) which may affect the movement of the tip and therefore incontestably proves the magnetic origin of the contrast. In our previous studies, we demonstrated the in situ presence of a small amount of BaFO magnetic phase in PLD synthesized thin film of TTB-Eu; nevertheless the amount of BaFO was too small to be able to detect a magnetic signal using MFM measurement [30,37]. Combining our previously published results with the present findings (Figure 5b), we conclude that the observed magnetic signal is due to the magnetic thin layer of BaFO deposited underneath the TTB-Eu ferroelectric film.

In order to further characterize the magnetic properties of our material, the variation of the macroscopic magnetization (M) was studied versus an applied magnetic field H as shown in Figure 5d. The hysteresis behavior of the magnetization clearly evidences the ferromagnetic nature of the heterostructure. In addition, the high value of the magnetic coercive field (H_C_ ≈ 2550 Oe) was consistent with the hard magnetic character of barium hexaferrite BaFO [49,59,60]. Similar magnetic behavior was also determined for TTB-Nd/BaFO thin films stacks synthesized by sputtering [61]. It has to be noted, however, that the magnetization at saturation measured in the synthesized heterostructure reported here was much higher compared to what we previously reported in nanocomposite TTB-Eu/BaFO, where the BaFO magnetic phase was in the form of nanoparticles embedded in the TTB-Eu ferroelectric matrix [30,37,39]. The excellent ferroelectric as well as magnetic properties demonstrated in the TTB-Eu/BaFO heterostructure unequivocally proves the room temperature multiferroic nature of the synthesized TTB-Eu/BaFO bilayer system.

### 3.3. Magnetoelectric Coupling

After demonstrating the occurrence of simultaneous ferroelectric and magnetic behavior at room temperature, we investigated whether the two properties were coupled. The coupling was tested both by looking at the effect of a magnetic field on the ferroelectric domain structure, and by observing the change in the magnetic domain contrast upon changing the electrical polarization by the application of a bias voltage.

**Figure 6:**
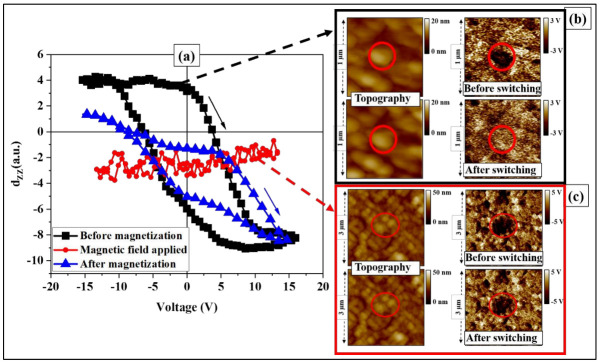
Suppression of ferroelectric switching by applying an external magnetic field: (**a**) piezoelectric hysteresis loops (d_ZZ_ vs. V) measured before (black), during (red) and after (blue) applying a magnetic field (≈2700 Oe). The image (**b**) shows the topography and the out-of-plane piezoelectric signal (z-PFM) before and after switching of the polarization (no magnetic field is applied). The image (**c**) shows the topography and the z-PFM signal before and after applying 15V bias voltage in presence of the applied magnetic field, showing no switching of the polarization. The red circles indicate the variation of the contrast upon poling before and after applying the external magnetic field, confirming the magnetoelectric effect.

In the first stage, we studied the variation of the local ferroelectric polarization, before, during and after applying a magnetic field as depicted in Figure 6. The black PFM loop, recorded in the absence of a magnetic field, shows a very clear hysteresis of the d_ZZ_ (V) dependence. Furthermore, the change in the contrast observed in the z-PFM images at the location on the sample where the bias was applied, before and after poling (see, encircled regions in Figure 6b) evidences the switching behavior of the ferroelectric polarization, which is consistent with the previous discussion and Figure 4. In addition, we checked for the presence of switching at several more locations, randomly chosen on the sample surface, and always found a similar hysteresis (see Figure S3 in Supplementary Materials).

Next, we applied a magnetic field of 2700 Oe (higher than the measured coercive magnetic field H_C_ of the BaFO layer, see Figure 5c), parallel to the surface of the sample and we measured again the ferroelectric switching. The red curve in Figure 6a shows that upon cycling the bias voltage under an applied magnetic field of 2700 Oe, the piezoelectric coefficient did not change its sign anymore (in contrast to the black curve), thus suggesting that the presence of a magnetic field suppresses the ability to switch polarization by applying an electric field. This result clearly demonstrates a magnetoelectric coupling, which takes the form of a suppression of the ferroelectric switching behavior by applying a magnetic field. Given the local character of this result, we repeated the experiment several times, at different locations on the sample surface, and with different AFM tips. We obtained similar results in each case (Figure S3 in Supplementary Materials), which demonstrates the reproducibility of the experiment and further confirms the presence of the magnetoelectric coupling across the heterostructure. Furthermore, the suppression of ferroelectric switching can also be seen in Figure 6c, which shows that the piezoelectric domain contrast does not change anymore upon poling, as opposed to the switching observed before applying the magnetic field (Figure 6b). The reversibility of the quenching of the magnetoelectric coupling was investigated by recording d_ZZ_ vs. V hysteresis loops after removing the external magnetic field. As shown by the blue curve (hysteresis loop) in Figure 6a, the switching of the polarization was restored, albeit showing different characteristics from the initial hysteresis (black curve). For instance, the blue loop exhibits a noticeable asymmetry, which can be explained by the effect of the residual strain induced by the remnant magnetization of the BaFO. This confirms that the application of an external magnetic excitation (field) is responsible for the suppression of the hysteresis loops, namely the switching character of the ferroelectric polarization. Thus the ferroelectric properties of the studied system can be controlled by an external magnetic excitation, which confirms the ‘direct’ magnetoelectric coupling.

**Figure 7:**
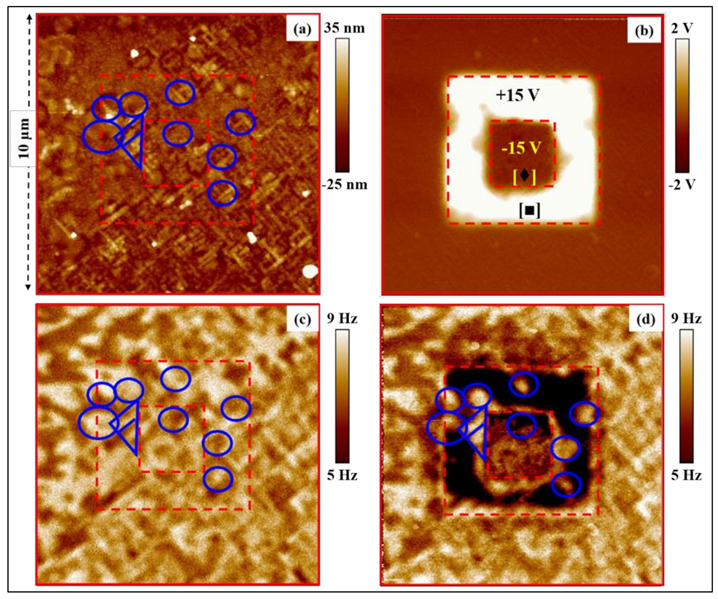
The influence of an applied voltage on the magnetic domains of the TTB-Eu/BaFO heterostructure after poling 5 × 5 µm^2^ with +15 V, then 2 × 2 µm^2^ with −15 V (see dotted contours). (**a**) Topography of surface of TTB-Eu/BaFO heterostructure. (**b**) Surface potential (KPFM) image, having the additional benefit of compensating for the electrostatic effects that could be present during the MFM measurements (see text). (**c**) and (**d**) are the MFM images, i.e., represent the magnetic response (frequency shift) of the heterostructure, respectively, before and after applying the DC electric poling excitation of ±15 V. The regions marked with blue circles and triangles show changed magnetic contrast. [■] and [♦] indicate regions with a negative and a positive frequency shift, respectively.

In the second stage, we investigated the effect of the polarization switching on the magnetic domain structure (i.e., the reverse magnetoelectric coupling) by performing MFM measurements before and after poling/switching the ferroelectric domains as depicted in Figure 7. The topography and the MFM contrast before the poling experiment are shown in Figure 7a,c, respectively. A KPFM image, recorded simultaneously, revealed an average surface potential of 0.14 V with a FWHM of its distribution of 30 mV (Figure S4 in Supplementary Materials). Then a square of 5 × 5 µm^2^ was scanned in contact mode with a bias of +15 V followed by a second scan of 2 × 2 µm^2^ with the tip biased at −15 V. Finally, an MFM measurement was again performed and the result is displayed in Figure 7d. In a previous study on multiferroic bilayer composites [62], we observed that the regions where a bias voltage had been applied also exhibited a strong change in the magnetic contrast (at locations where magnetic domains were not expected), and we associated this change to the electrostatic interaction (caused by polarization switching and/or injected charges) which also affects the resonance frequency shift used to detect the magnetic domains in MFM [63,64]. Recently, several authors suggested different methods to distinguish between magnetic and electrostatic interactions in MFM [65,66,67,68,69]. Among these methods, we argue that the most appropriate approach is to continuously compensate the electrostatic interaction, (including during the topography measurement) since it can affect the resonance frequency (thus both the topography and the MFM signal). Practically, the electrostatic compensation is already achieved in typical (closed-loop) KPFM, since the surface potential measurement in this method is based precisely on the minimization of the electrostatic force between tip and sample. Therefore, the MFM presented here was performed simultaneously with KPFM in order to eliminate the electrostatic artifacts. As can be seen in Figure 7b, the surface potential changed drastically following the poling scan, a fact which we attribute to a combination of both polarization switching and charge injection. Noteworthy, the topography image is identical to that observed before the poling (Figure S5 in SI), thus suggesting that no chemical or structural modification of the surface occurred. The MFM image (Figure 7d) also shows significant changes within the area poled, compared to the initial magnetic domains (Figure 7c). Remarkably, the area marked [■] (the region poled with +15 V outside the inner square) shows a negative frequency shift of about 7 Hz compared to the average frequency shift measured across the surface. We believe that the contrast between these regions is due to a residual electrostatic artifact and does not represent changes in the magnetic domain structure. Indeed, the electrostatic compensation performed is based on the amplitude-modulated KPFM (AM-KPFM), which, as pointed out by several authors, is known to suffer from poorer spatial resolution as compared to the frequency-modulated KPFM (FM-KPFM) [46,65], implying that the compensation of the electrostatic force is not perfect. Ideally, the compensation should be performed using FM-KPFM, but the latter cannot be implemented in this case because it would nullify the total frequency shift, including the shift caused by the magnetic interactions. However, careful comparison between Figure 7c,d shows that the contrast at several locations (marked with blue contour lines) has changed following the poling procedure, and the differences [66] can only be explained by changes in the local magnetization. These changes in the magnetic domain structure following the poling procedure suggests that applying an electric field modifies not only the ferroelectric polarization, but also the magnetization in the studied multiferroic heterostructures, which hints at a reverse magnetoelectric coupling.

## 4. Discussion

The magnetoelectric coupling in our heterostructured system is the result of the large strain-mediated interactions at the interfaces between the magnetic layer (BaFO) and the ferroelectric layer (TTB-Eu) due to the epitaxial relationship between the two components, as discussed above. The magnetoelectric effect via the elastic coupling transmitted through the epitaxially strained interface is very well identified and studied for magnetoelectric composite films [6,10,70]. Indeed, by applying a magnetic field, the magnetic component undergoes a mechanical deformation via the converse piezomagnetic (or magnetostrictive) effect. Consequently, the ferroelectric component–strongly elastically coupled at the interface due to the good matching and lattice continuity of the two crystallographic phases–is subject to a mechanical stress transmitted from the magnetic component. Typically, this applied stress will change the polarization of the ferroelectric component via the piezoelectric (or electrostrictive) effect, as depicted in Figure 6 [10,52,70,71]. In our case, the effect of the stress is to reduce the polarization switching, similar to the clamping of films on a substrate [72]. Conversely, the application of an electric excitation causes a deformation of the ferroelectric component (via the inverse piezoelectric effect). This deformation is transferred to the magnetic phase through the epitaxially strained interface between TTB-Eu and BaFO phases, resulting in a change of the magnetic properties, in our case the domain structure of the BaFO phase, as shown in Figure 7. This direct and reverse magnetoelectric coupling in the TTB-Eu/BaFO heterostructures could even be strengthened by the possible lateral epitaxy between the two phases (yellow lines in Figure 3d).

## 5. Conclusions

High-quality Ba_2_EuFeNb_4_O_15_/BaFe_12_O_19_ (TTB-Eu/BaFO) epitaxial bilayered heterostructures have been grown on 001-oriented Nb-doped SrTiO_3_ substrates (NSTO (001)) by PLD. The epitaxial nature as well as the relationships between the NSTO substrate and the two TTB-Eu and BaFO phases were established with X-ray diffraction. The microstructure was studied using scanning force microscopy (SPM/AFM). The ferroelectric properties – originating from the TTB-Eu component – were demonstrated by investigating the microelectromechanical properties of the synthesized composite using piezoresponse force microscopy (PFM). The magnetic properties were demonstrated both at the microscopic and macroscopic scales by visualizing magnetic domains using magnetic force microcopy (MFM) and by showing hysteretic switching of the macroscopic magnetization using vibrating sample magnetometer (VSM). The obtained results are consistent with the known magnetic properties of the BaFO component. Moreover, a strong magnetoelectric coupling at room temperature was demonstrated in both directions: first, we showed that an applied magnetic field changes the ferroelectric properties by suppressing the polarization switching. Then, we demonstrated the influence of an applied electric field on the magnetic domain structure of the nanocomposite (reverse magnetoelectric effect). Thus, we achieved the synthesis of high-quality TTB-Eu/BaFO nanocomposite bilayered heterostructures, which exhibited room temperature multiferroic properties as well as a clear magnetoelectric coupling.

## Data Availability

Not applicable.

## Supplementary Materials

The following supporting information can be downloaded at: https://www.mdpi.com/article/10.3390/nano13040761/s1, Figure S1: Topography of BaFO thin films (thickness≈20 nm) deposited on NSTO (001) substrates according to the deposition conditions specified in the main manuscript: (a) as deposited thin films, (b) films annealed after deposition at 850 °C and (c) films annealed a second time at 800 °C, showing that the second annealing does not alter the films microstructure; Figure S2: Schematic illustration of TTB-Eu and BaFO structures on NSTO substrate in the case where the (111) diffraction peaks of NSTO (i.e., (111) plane) are aligned with the (107) diffraction peaks of BaFO (i.e. (107) plane) and which are rotated by 45° with respect to the diffraction peaks of (221) planes of TTB-Eu structure; Figure S3: Variation of the longitudinal piezoelectric coefficient (dZZ) as a function of an applied voltage (a) in the absence and (b) in the presence of an applied magnetic excitation measured at different locations on the studied heterostructured films TTB-Eu/BaFO; Figure S4: (a) KPFM image measured simultaneously with the topography and MFM image before poling (i.e., simultaneously with the image 7c in the main manuscript). (b) Distribution of the surface potential; Figure S5: comparison of the topography of the TTB-Eu/BaFO heterostructure (a) before and (b) after applying the electric excitation shown in Figure 7 in the main manuscript.
