# Supplementary material for: Magnetoelectric Coupling in Room Temperature Multiferroic Ba2EuFeNb4O15/BaFe12O19 Epitaxial Heterostructures Grown by Laser Ablation
Source: Nanomaterials (Basel). 2023 Feb 17;13(4):761. doi: 10.3390/nano13040761 (PMC9967026; doi:10.3390/nano13040761)
Supplement: Supplementary file 1 [file nanomaterials-13-00761-s001.zip › nanomaterials-2197636-supplementary.pdf]

S1: evolution of topography of BaFO films as a function of temperature

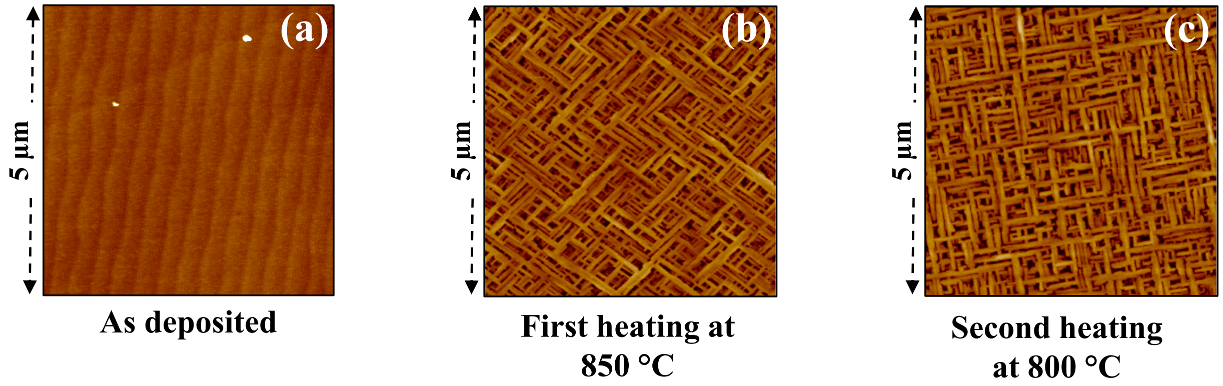

Fig. S1: Topography of BaFO thin films (thickness ≈ 20 nm) deposited on NSTO(100) substrates according to the deposition conditions specified in the main manuscript: (a) as deposited thin films, (b) films annealed after deposition at 850 °C and (c) films annealed a second time at 800 °C, showing that the second annealing does not alter the films microstructure.

The comparison of (a) and (b) shows the influence of the annealing after deposition at 850 °C on the formation of the desired microstructure of BaFO/NSTO(100) thin films. The comparison of (b) and (c) demonstrates that the second annealing at 800 °C does not affect the BaFO films microstructure. This result proves that when the system TTB-Eu/BaFO is annealing at 800 °C, this temperature allows crystallizing the TTB-Eu component without affecting the BaFO microstructure.

S2: illustration of epitaxial growth

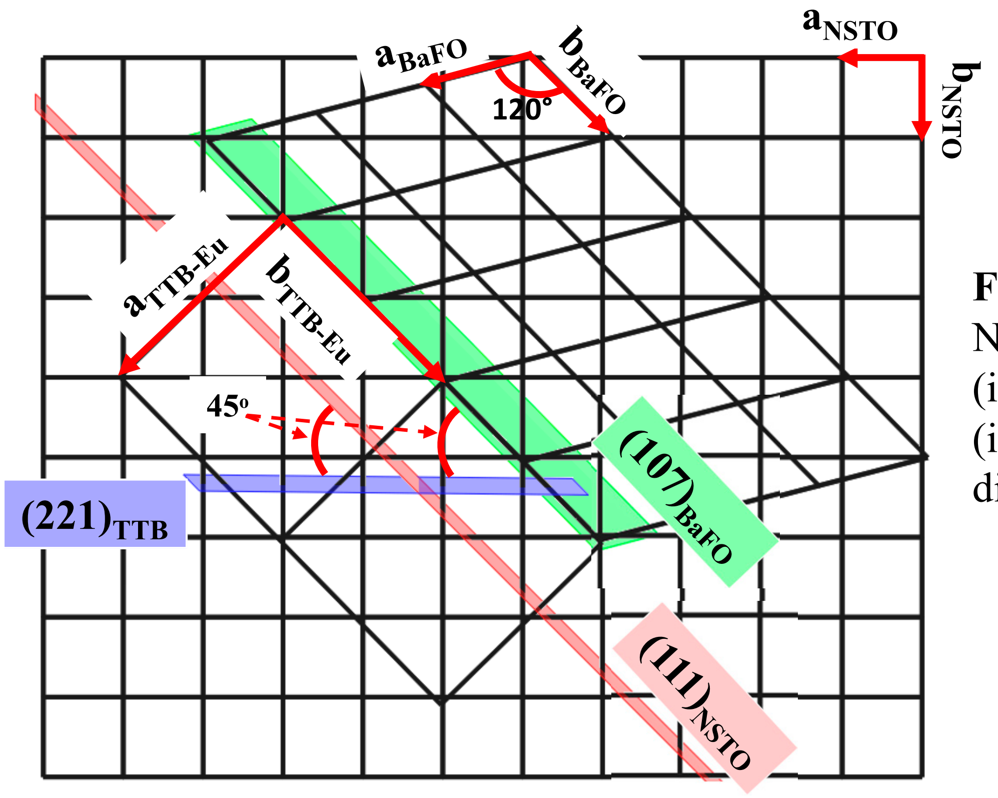

Fig. S2: Schematic illustration of TTB-Eu and BaFO structures on NSTO substrate in the case where the (111) diffraction peaks of NSTO (i.e. (111) plane) are aligned with the (107) diffraction peaks of BaFO (i.e. (107) plane) and which are rotated by 45° with respect to the diffraction peaks of (221) planes of TTB-Eu structure.

S3: magnetoelectric coupling

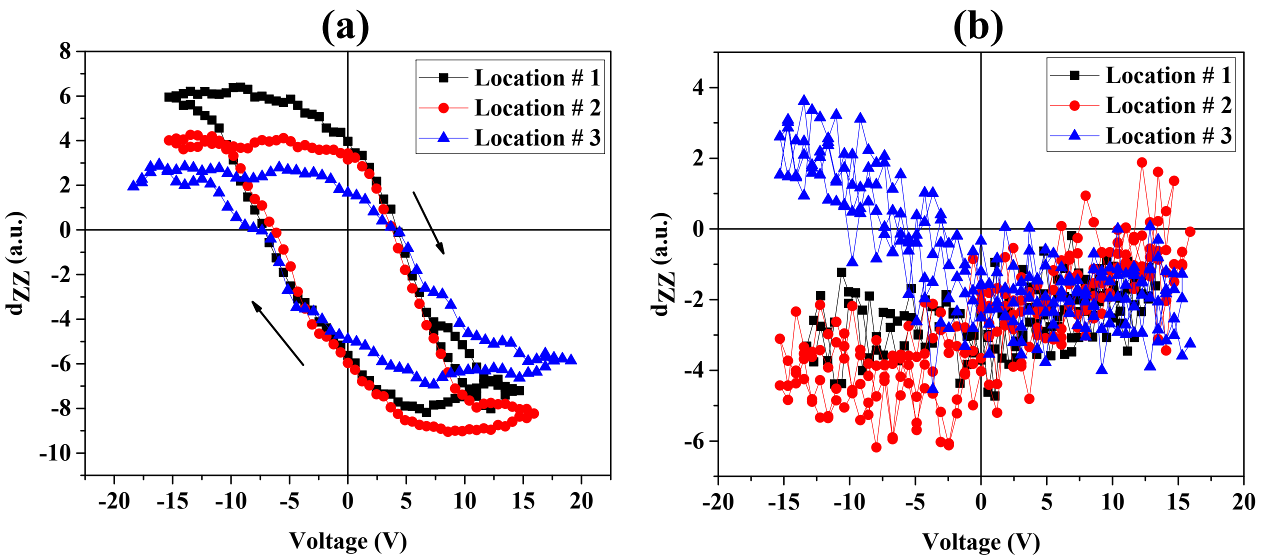

Fig. S3: Variation of the longitudinal piezoelectric coefficient (d_{ZZ}) as a function of an applied voltage (a) in the absence and (b) in the presence of an applied magnetic excitation measured at different locations on the studied heterostructured films TTB-Eu/BaFO.

As discussed in the main manuscript, an applied magnetic field can tune the ferroelectric properties, evidencing the presence of a magnetoelectric coupling in the synthesized multiferroic (ferroelectric/ferromagnetic) system TTB-Eu/BaFO. In the **Fig. S3**, we show the influence of the applied magnetic field on the hysteresis behavior of d_{ZZ} vs. V (the ferroelectric properties) obtained at different locations on the samples. This verification proves the reproducibility of the measurement and confirms the magnetoelectric coupling uniformly present in the synthesized (TTB-Eu/BaFO) heterostructure.

S3: magnetoelectric coupling

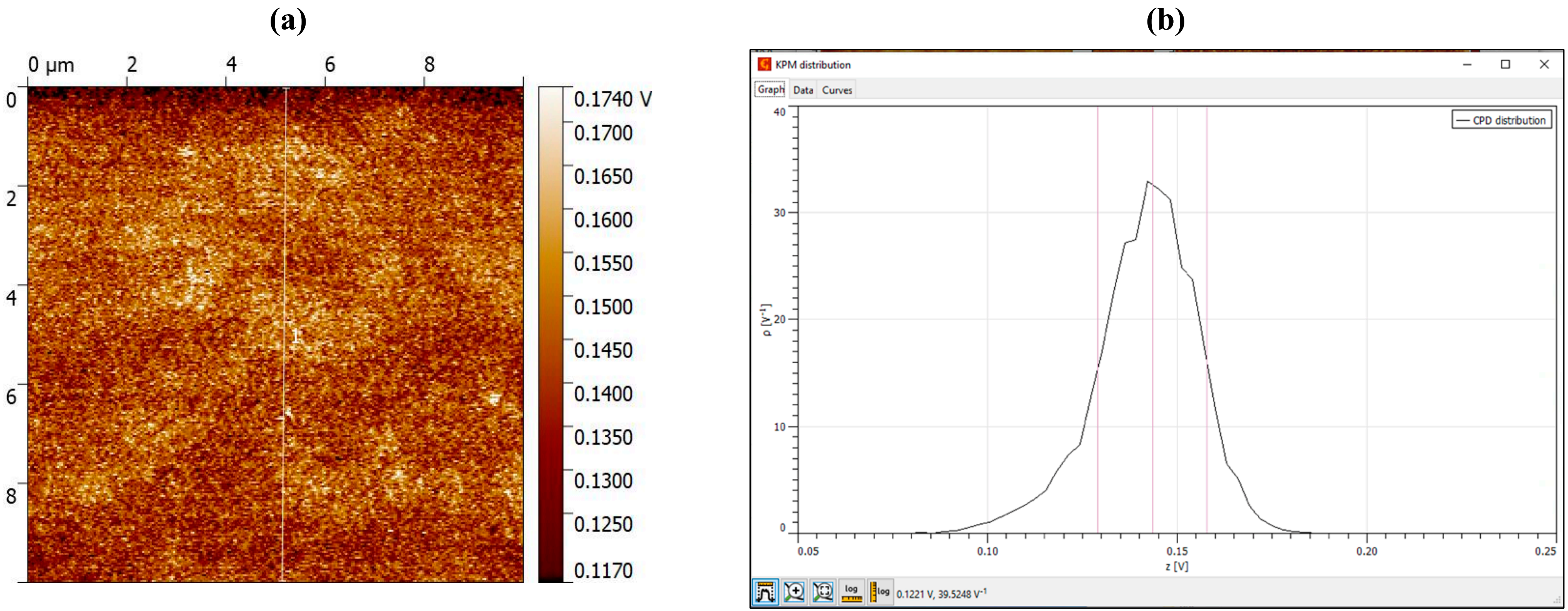

Fig. S4: (a) KPFM image measured simultaneously with the topography and MFM image before poling (i.e. simultaneously with the **image 7(c)** in the main manuscript). (b) Distribution of the surface potential.

S3: magnetoelectric coupling

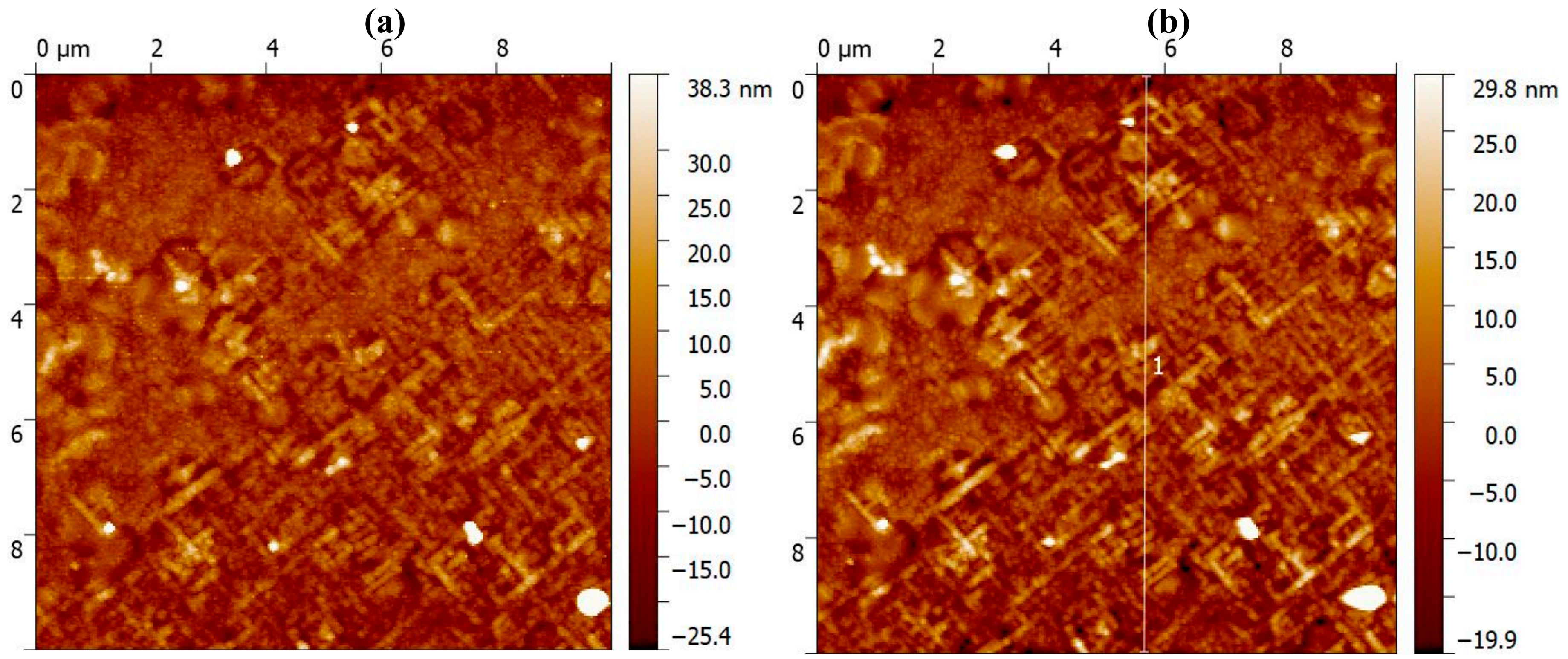

Fig. S5: comparison of the topography of the TTB-Eu/BaFO heterostructure (a) before and (b) after applying the electric excitation shown in **Figure 7** in the main manuscript.
